# Bridging the Research–Practice Gap: Diffusion and User Experience of a Digital Health Platform in Swedish Athletics

**DOI:** 10.1155/tsm2/2452118

**Published:** 2026-09-30

**Authors:** Jenny Jacobsson, Per Nilsen, Siw Carlfjord, Jan Kowalski, Örjan Dahlström, Ulrika Tranaeus

**Affiliations:** ^1^ Department of Health, Medicine and Caring Sciences Linköping University Linköping Sweden liu.se; ^2^ Athletics Research Center Linköping University Linköping Sweden liu.se; ^3^ Swedish Athletics Federation Stockholm Sweden; ^4^ Department of Behavioural Sciences and Learning Linköping University Linköping Sweden liu.se; ^5^ Department of Physiology, Nutrition and Biomechanics The Swedish School of Sport and Health Sciences GIH Stockholm Sweden gih.se; ^6^ Sport Performance & Sport Medicine GIH Stockholm Sweden

**Keywords:** digital health, implementation, prevention, sports medicine, user experience

## Abstract

**Background:**

Evidence‐based health information often fails to reach everyday sports practice, leaving a gap between research and real‐world application. Digital platforms can help bridge this research–practice gap by disseminating accessible and sport‐specific health guidance to coaches, parents, and athletes. *Frisk Friidrott*, developed by the Swedish Athletics Federation, is a digital health platform providing evidence‐based information on injury prevention, training, nutrition, and mental health in youth athletics.

**Objective:**

To evaluate the diffusion and user experience of *Frisk Friidrott* among one group of intended end users, coaches and parents, for youth aged 12–15 years in Swedish Athletics.

**Methods:**

A multimethods, cross‐sectional study was conducted among coaches and parents of athletes aged 12–15 years. Quantitative data (*n* = 267) were collected through a web‐based survey and analyzed descriptively. Qualitative data were obtained from open‐ended survey responses (*n* = 40) and semistructured interviews (*n* = 5) and analyzed thematically. Two dimensions from the Reach, Effectiveness, Adoption, Implementation and Maintenance Sports Setting Matrix (RE‐AIM‐SSM framework), reach and adoption, were used to evaluate diffusion, while constructs from the Technology Acceptance Model, perceived usefulness and perceived ease of use, were applied to assess user experience.

**Results:**

Thirty‐one percent of respondents had heard of the platform, and 27% had initially adopted it. Adoption was higher among coaches, especially those with training in sports medicine or athletics. Adopters reported high perceived usefulness and credibility but identified navigation and structural limitations as barriers to ease of use.

**Conclusion:**

Passive diffusion resulted in modest reach. Integrating the platform into coach education and improving usability and promotion may enhance adoption and sustained engagement within Swedish Athletics.

## 1. Introduction

Protecting and promoting the health of athletes requires a holistic approach that integrates multiple strategies, including appropriate physical conditioning, balanced nutrition, safe and well‐fitted equipment, and adherence to preventive routines such as proper warm‐ups and cool‐downs [1, 2]. Despite substantial advances in sports medicine research, translating scientific knowledge into everyday sports practice remains a challenge. The so‐called research–practice gap persists because accessible, context‐specific, and evidence‐based information about athlete health and injury prevention is often lacking within the sporting environment [3].

Digitalization offers new possibilities for reducing this gap. With the increasing use of apps, websites, and other digital tools, functions traditionally provided offline can now be delivered more efficiently and at low cost. Within sport, digital media can serve as a simple yet powerful means of disseminating relevant health information, enabling federations and clubs to communicate evidence‐based knowledge directly to their members [4, 5].

In response to this potential, the Swedish Athletics Federation developed *Frisk Friidrott* (https://www.friidrott.se/frisk-friidrott/), a digital health platform that compiles current evidence‐based knowledge related to health and training in athletics [6]. The first version of the platform, launched in 2019, contained health information for athletes aged 12–15 and aimed to bridge the research–practice gap by providing coaches and parents with easily accessible, athletics‐specific health guidance. The site includes information on common injuries and their management, nutritional recommendations, mental health, recovery, and athlete growth and development.

The platform’s effectiveness has previously been demonstrated in a cluster‐randomized controlled trial, which showed a 50% reduction in injury incidence among athletes in the group where coaches and parents had access to the platform compared with controls [7]. These findings suggest that sport‐specific, digital, health‐promoting tools can be an effective complement to established injury prevention and health support programs [8]. However, promising results in controlled research settings do not automatically translate into sustainable real‐world use. Many evidence‐based prevention programs struggle to achieve long‐term adoption or integration into everyday sports practice [9, 10]. The dissemination of digital media—such as health platforms—within sports environments has received limited scholarly attention [11, 12].

Implementation science studies the challenges of the research–practice gap by evaluating strategies for translating research into routine practice, ultimately supporting actions to improve healthcare quality and population health. [13]. Within this field, *dissemination* refers to the active and planned spread of innovations to a target audience using strategic communication approaches [14, 15]. In contrast, *diffusion* describes the more passive, unplanned spread of new practices as they are gradually adopted within a community or social system [14]. Understanding both processes is essential to improving the reach and longevity of evidence‐based sports health initiatives.

Rogers’ The Diffusion of Innovation theory [16] offers a useful lens for examining how innovations such as *Frisk Friidrott* spread and are adopted. According to this theory, the diffusion process is influenced by the characteristics of the innovation itself, the traits of its adopters, the structure of the social system, and the communication channels through which information is shared. Building insights around these components can inform tailored dissemination strategies, helping innovations reach and engage a broader audience [17, 18].

A central factor in diffusion is the user experience, as individuals’ perceptions of an innovation strongly influence their willingness to adopt and continue using it [19]. Rogers identifies five key innovation attributes, i.e., relative advantage, compatibility, complexity, trialability, and observability, that shape adoption behavior [16]. In the context of digital health, these correspond closely to the concepts of *perceived usefulness* and *perceived ease of use* from the Technology Acceptance Model [20, 21]. Both are crucial determinants of technology acceptance and sustained engagement [22].

To date, the *Frisk Friidrott* platform has primarily spread within Swedish Athletics through passive diffusion rather than active dissemination. The primary aim of this study was therefore to evaluate the diffusion of the digital health platform within Swedish Athletics, focusing on one group of intended end users of the health platform, coaches and parents/caregivers, of youth athletes aged 12–15 years. Specifically, the study examined two dimensions of the Reach–Effectiveness–Adoption–Implementation–Maintenance Sports Setting Matrix (RE‐AIM‐SSM) framework: reach and adoption [23]. A secondary aim was to assess adopters’ user experience, including their perceived usefulness and perceived ease of use of the platform.

## 2. Methods

### 2.1. Study Design

Quantitative data were collected through a cross‐sectional survey, complemented by additional qualitative data from open‐ended survey responses and semistructured interviews [24].

Two dimensions of the RE‐AIM‐SSM framework, i.e., reach and adoption, were analyzed to evaluate the diffusion of the digital health platform (Table 1) [15, 25]. The selection of these two dimensions was based on their direct relevance to studying diffusion rather than active implementation. Reach reflects the extent to which an innovation becomes known to its intended audience, indicating the breadth of exposure within a social system, while adoption represents the proportion of individuals or organizations that take up and begin using the innovation in practice. Accordingly, adoption was in this context operationalized as at least one visit to the platform, reflecting initial uptake of the innovation at the individual level [13].

**TABLE 1:** Definitions of outcomes examined in the study [10, 18].

	Operational definitions
Reach	The number and proportion aware of the health platform by category (“parent and coach,” “parent only,” “coach only”)

Adoption	The number and proportion who have visited the health platform by category (“parent and coach,” “parent only,” “coach only”)
	The number and proportion who report visiting (using) the health platform (Frisk.Friidrott) and intention to use it in planned activities and/or their practice by category (“parent and coach,” “parent only,” “coach only”)

Perceived usefulness	The adopters’ perception that using the digital health platform (Frisk.Friidrott) will enhance job performance, a key factor influencing adoption of technology

Perceived ease of use	The adopters’ perception that using the digital health platform (Frisk.Friidrott) requires minimal effort, which affects attitudes toward adoption

Because the *Frisk Friidrott* platform had not yet been actively disseminated at the national level, later RE‐AIM stages, such as implementation fidelity or maintenance, were outside the study’s analytical scope. Focusing on reach and adoption therefore allowed a pragmatic and theoretically consistent evaluation of how far the platform had diffused and which user groups had engaged with it during the early, largely unassisted phase of its lifecycle.

To explore user experience, two constructs from the Technology Acceptance Model, i.e., perceived usefulness and perceived ease of use, were examined, as these are central predictors of the acceptance and continued use of web‐based information systems (Table 1) [20, 21]. Perceived usefulness reflects the degree to which users believe the system enhances their work or activities, while perceived ease of use indicates how effortless the system is to operate [22]. These two constructs were selected because they form the core determinants of technology acceptance within the Technology Acceptance Model. Together, they capture the main perceptual drivers of adoption and sustained engagement observed across digital health and web‐based systems [22]. Given that the *Frisk Friidrott* platform is used voluntarily by coaches and parents outside formal healthcare structures, these two dimensions offered a parsimonious and theory‐based means of understanding how user perceptions influence diffusion within a community sport context.

A multimethods design was selected to capture both the extent and the context of diffusion. The qualitative component of this study was designed to complement the quantitative findings by providing richer, more contextualized insights into two key areas: first, the perceived usefulness of the health platform among its targeted end users and second, to identify suggestions for improvements from those who had adopted the website, with the goal of supporting sustained engagement and continued use over time [17]. The combination of quantitative and qualitative data allows for triangulation between measurable outcomes and user‐reported experiences, thereby offering a more comprehensive understanding of how the platform was accessed, adopted, and perceived by targeted end users in this study [13, 23].

The cross‐sectional component of the study was designed and reported in accordance with the Strengthening the Reporting of Observational Studies in Epidemiology (STROBE) statement [25]. The qualitative component followed the Consolidated Criteria for Reporting Qualitative Research (COREQ) guidelines [26] (Online Supporting Information Flow Charts (available here).

### 2.2. Creation and Dissemination Initiatives of the Digital Health Platform

The digital health platform *Frisk Friidrott* was developed in 2017 by Swedish Athletics in collaboration with intended end users and domain experts in medicine, physiotherapy, nutrition, and related fields [6]. The first version of the platform contained health information for children in early puberty, aged 12–15 years. The platform was kept internal during its development and the preceding randomized controlled trial [7]. It was publicly launched in April 2019, accompanied by a press release from Swedish Athletics and the creation of an Instagram account managed by the research group. No other active implementation or dissemination strategies were initiated between 2019 and autumn 2022.

Since 2019, the health platform has been further developed and now offers health information for athletes over 16, along with targeted content for specific groups of track and field athletes—such as female health and master athletes. In autumn 2022, the head researcher (JJ) was contacted by the Swedish Athletics Department of Education regarding the formal integration of the digital platform into national coach education programs.

### 2.3. Website Access

During the period from January 1 to December 31, 2022, Google Analytics recorded 30,946 sessions on the digital health platform *Frisk Friidrott* (https://www.friidrott.se/frisk-friidrott/), with an average session duration of 2.37 min. The majority of users reached the site either through Google search or by direct access. The most engaging pages on the platform were those covering “training during puberty,” “common injuries in athletics,” and “illnesses.”

### 2.4. Participants and Recruitment

Athletics in Sweden is organized according to the Scandinavian sports model, nonprofit and community‐based, and governed by the Swedish Athletics Federation, under the Swedish Sports Confederation [27]. The federation is responsible for overarching national operations, including education, competition, marketing, and national teams. Below the national level are nine regional districts (as of 2022), supporting local athletics clubs, whose activities are largely run by volunteers. As there is no licensing system for coaches or athletes, particularly within children’s and youth athletics, reaching intended target groups for data collection is challenging.

To maximize recruitment among parents and coaches of athletes aged 12–15 years, three complementary strategies were used:

1. Elite Clubs Association outreach: The 21 clubs within the Elite Clubs Association were invited between April and October 2023 via email and telephone by one of the authors (JJ). These clubs were selected because of their participation in earlier research and their established collaboration with the federation. The 21 clubs are well spread across Sweden, run larger athletics activities (e.g., arrange competitions and races) with relatively good opportunities to reach them as most have offices with employed staff. A meeting between JJ and the Association’s chairperson in July 2023 facilitated contact with relevant club representatives. In October 2023, all clubs received written information and an invitation to participate. Due to GDPR, the research team could not directly contact potential participants, and clubs were therefore asked to forward the invitation to eligible coaches and parents.
2. Social media dissemination: Smaller clubs do not have clear contact channels, so social media was chosen as another strategy. To reach a broader sample from smaller clubs, study information was shared on Swedish Athletics’ official Instagram and Facebook platforms in November 2023.
3. Coach education network: In January 2024, Swedish Athletics’ Department of Education emailed invitations to approximately 70 coaches who had completed the central youth coach education program (for ages 14–17) between 2019 and 2022.

For the semistructured interviews, we invited participants among the respondents of the survey who had reported that they had adopted the digital health platform and expressed interest in being interviewed (*n* = 9). Those who consented received follow‐up invitations via email between January and April 2024.

### 2.5. Data Collection and Analysis

Data were collected from November 2023 to March 2024 through a web‐based survey and semistructured interviews. The survey, administered via Survey&Report, was adapted from a previous football study [12] and included items from the System Usability Scale [28], revised to fit the athletics context (see Online Supporting Information A).

The survey instrument incorporated items measuring perceived usefulness and perceived ease of use, based on the Technology Acceptance Model [20, 21]. Face and content validity was established through expert review by five athletics coaches and two researchers in sports medicine, who tested the questionnaire for relevance, clarity, and item formulation. Minor linguistic adjustments were made following feedback. Internal consistency was acceptable for both constructs (Cronbach’s *α* > 0.80 for perceived usefulness and > 0.75 for perceived ease of use), indicating good reliability within the present sample [21, 22].

The survey’s first section captured demographic information (age, gender, and role in sport) and items assessing the platform’s reach (Table 1). The second section, directed toward respondents who had adopted the platform, contained items on adoption and user experience, including open‐ended questions for qualitative responses.

Qualitative data were gathered from (1) four open‐ended questions in the survey (*n* = 40) and (2) semistructured interviews (*n* = 5) conducted via Microsoft Teams by JJ. All interviews followed the same guide (see Online Supporting Information B), lasted 45–60 min, and began with a brief introduction and informed consent. Participants were asked to reflect on how they sought health information in youth sport and their experiences using the *Frisk Friidrott* platform. Interviews were audio‐recorded on a secure application, transcribed verbatim, and reviewed by two researchers (JJ and UT). Audio files were deleted after transcription. In this exploratory nature of the study, we used a limited number of adopters that were willing to participate, as using a thematic analysis saturation was not needed [29].

Qualitative data from free‐text survey responses and interview transcripts were integrated and analyzed using a hybrid deductive‐inductive thematic analysis according to Braun and Clarke [30]. To ensure rigor, two researchers (UT and JJ) independently coded the dataset within NVivo 1.7 (QSR International). The coding process followed a two‐phase protocol. First, a deductive approach was applied using a provisional codebook containing three predefined theoretical categories: diffusion, usefulness, and ease of use. Second, an inductive approach was employed to identify data‐driven subcodes directly from the text. Intercoder agreement exceeded 90% during the independent coding phase. Discrepancies were resolved through collaborative discussion until 100% consensus was achieved. Finalized subcodes were synthesized into overarching themes and interpreted in direct relation to the study’s theoretical framework.

### 2.6. Statistical Analysis

As this was an exploratory study, no formal sample size calculation was performed. Participants were categorized into three groups, “parent and coach,” “parent only,” and “coach only,” to reflect ecological roles within community sport [1, 27]. Quantitative data from the cross‐sectional survey are presented descriptively using frequencies and proportions for categorical variables and means ± standard deviations for continuous variables.

Recruiting the intended participants for this study proved challenging, as Swedish child and youth sports rely primarily on volunteer administrators. Consequently, the total number of individuals who received the study invitation is unknown and a response rate could not be calculated. Among respondents, missing data were minimal and limited to a small number of individual questionnaire items. The number of valid responses for each variable is reported in the tables. Given the very low proportion of missing data, no imputation procedures were applied, and analyses were conducted using the observed data only.

Differences between adopters and nonadopters for the variables sex, former athlete (yes/no), previous coach education in athletics (yes/no), coach program in other sports (yes/no), and previous attendance to education in sports medicine (yes/no) were assessed using chi‐squared tests. Differences in age were tested using the *t*‐test. All analyses were two‐sided at the 0.05 level of significance and performed in IBM SPSS version 28.0.

## 3. Results

### 3.1. Demographic Information of Survey Respondents

Of the 21 elite clubs contacted, six confirmed that they would forward the study invitation to their members. For the remaining clubs, no information was received regarding their response to the mailing. The final survey sample (*n* = 267) represented all nine regional districts. In total, 267 individuals completed the survey; 23% (*n* = 61) were both coach and parent of athletes, 46% (*n* = 124) were parent only, and 31% (*n* = 82) were coach only (Table 2). The mean age of all respondents was 46 years (range 16–65), and 54% (*n* = 143) were female. For the semistructured interviews, five responded and were interviewed (men *n* = 4, coach and parent *n* = 4, and coach only *n* = 1, no information about age was collected).

**TABLE 2:** Descriptive statistics of the responders.

	Coach and parent, *n* (%)	Parent only, *n* (%)	Coach only, *n* (%)
Participants (*N* = 267)	61 (23)	124 (46)	82 (31)
Age (years), mean (SD)	48 (4.9)	49 (5.5)	42 (13)
Sex, female	28 (46)	84 (68)	31 (38)
Country of birth			
Sweden	60 (98)	115 (92)	77 (94)
Norway, Finland, Denmark, Iceland	0 (0)	4 (3)	1 (1)
Other country in Europe	1 (2)	3 (2)	1 (1)
Country outside Europe	0 (0)	2 (2)	3 (4)
Former athlete			
Yes	49 (80)	80 (65)	74 (90)
No	12 (20)	44 (35)	8 (9)
Currently coaching			
Yes	50 (82)	7 (6)	70 (85)
No	11 (18)	117 (94)	12 (15)
Athletics as primary sport as coach	49 (98)	2 (29)	66 (97)
Any coach education in athletics^†^			
Yes	56 (93)	16 (13)	72 (89)
No	4 (7)	108 (87)	9 (11)
Level of coach education^†^			
Child	40 (71)	9 (56)	44 (62)
Youth 10–14 years	39 (70)	6 (38)	55 (78)
Youth 14–17 years	27 (48)	2 (13)	40 (56)
Certified coach	3 (5)	0 (0)	9 (13)
Coach program in another sport			
Yes	23 (38)	35 (28)	22 (27)
No	38 (62)	89 (72)	60 (73)
Does your club or your district offer any education in sports medicine?			
Yes	20 (33)	16 (13)	26 (32)
No	13 (21)	13 (10)	21 (26)
Do not know	28 (46)	95 (77)	35 (43)
Have you attended any education in sports medicine, e.g., tape, acute management?			
Yes	30 (49)	18 (14)	43 (52)
No	30 (49)	104 (84)	37 (45)
Do not know	1 (0)	2 (2)	2 (2)
How do you get hold of information about injuries in athletics, acute management, and nutrition?^‡^			
The web	56 (92)	105 (85)	65 (79)
Asking other coaches or parents	38 (62)	49 (39)	43 (52)
Contact your health center	2 (3)	24 (19)	12 (15)
Contact with doctor/physiotherapist	39 (64)	59 (47)	42 (51)
Other	9 (15)	8 (6)	15 (18)

^†^One coach and parent and one coach only did not respond to the question.

^‡^Could report more than one option.

Among all respondents, 76% (*n* = 203) reported having a background as athletes, and 54% (*n* = 144) had completed a coach education program in athletics, while 48% (*n* = 127) were currently active as coaches. Twenty‐three percent (*n* = 62) were aware of sports medicine courses arranged by their club or district, and 59% (*n* = 158) reported no such awareness. The internet was identified as the primary source of health‐related information by 85% (*n* = 226) of respondents, with the official Swedish health website 1177.se being the preferred platform for information and healthcare services.

### 3.2. Reach and Adoption of the Digital Health Platform

Among all respondents (Table 3), 31% (*n* = 84) had heard of the digital health platform. Among those, the primary source of information was Swedish Athletics 71% (*n* = 60), followed by clubs and other coaches 15% (*n* = 13) and district organizations 11% (*n* = 9). Overall, 27% (*n* = 73) of respondents reported having visited the platform, indicating initial adoption.

**TABLE 3:** Reach and adoption of the digital health platform.

	Coach and parent, *n* (%)	Parent only, *n* (%)	Coach only, *n* (%)
Reach (*N* = 267)	61 (23)	124 (46)	82 (31)
Have you heard of the digital health platform Frisk.Friidrott?			
Yes	32 (52)	10 (8)	42 (51)
No	29 (48)	114 (92)	40 (49)
How did you learn about the digital health platform Frisk.Friidrott?^†^ ^,^ ^‡^			
Swedish Athletics Federation (centrally)	25 (78)	4 (40)	31 (74)
My district	2 (6)	0 (0)	7 (17)
My club	2 (6)	2 (20)	9 (21)
Other coaches	6 (19)	1 (10)	6 (14)
Other parents	0 (0)	0 (0)	0 (0)
Doctor, physiotherapist	0 (0)	0 (0)	4 (10)
Other	6 (19)	4 (40)	8 (19)
Have you visited the digital health platform Frisk.Friidrott?			
Yes	27 (44)	9 (7)	37 (45)
No	34 (56)	115 (93)	45 (55)

^†^Answered by those that had heard of the digital health platform (*n* = 84).

^‡^Could report more than one option.

There were no significant differences between adopters and nonadopters with respect to sex, age, having a background as an athlete, or completion of a coach education program in another sport (Table 4). However, respondents who had attended at least one sports medicine course were approximately 2.3 times more likely to adopt the platform than those who had not, *p* < 0.001. Similarly, those with a coach education in athletics were twice as likely to have adopted the platform compared with those without such education but was not statistically significant, *p* = 0.10.

**TABLE 4:** Descriptive statistics of adopters and nonadopters of the digital health platform.

Participants, all, *n* (%)	Adopters *n* = 73 (27)	Nonadopters *n* = 194 (73)	*p* value	
Age (years), mean (SD)	43.6 (10.6)	45.4 (11.0)	0.23	(*t*‐test)
Parent	9 (7)	115 (93)		
Coach and parent	27 (44)	34 (56)		
Coach	37 (45)	45 (55)		

**Participants, coaches^†^, *n* (%)**	**64 (45)**	**79 (55)**		

Sex				
Female	26 (44)	33 (56)	0.89	(Chi‐square test)
Male	38 (45)	46 (55)		
Former athlete				
Yes	55 (45)	68 (55)	0.98	
No	9 (45)	11 (55)		
Any coach education in athletics^‡^				
Yes	60 (47)	68 (53)	0.10	
No	3 (23)	10 (77)		
Coach program in another sport^§^				
Yes	18 (40)	27 (60)	0.68	
No	46 (47)	51 (53)		
Have you attended any education in sports medicine, e.g., tape, acute management?				
Yes	45 (62)	28 (38)	< 0.001	
No	18 (27)	49 (73)		
Do not know	1 (33)	2 (67)		

*Note:* For the calculation of the chi‐square test comparing education in sports medicine, the category “No” and “Do not know” were pooled together.

^†^Including “coach and parent only” and “coach only.”

^‡^One adopter and one nonadopter did not respond to the question.

^§^One nonadopter did not respond to the question.

Among adopters (*n* = 73) and frequency of use, 15% (*n* = 11) reported visiting the platform monthly, 36% (*n* = 26) less often but regularly, and 48% (*n* = 35) only some rare occasions (Table 5). The most frequently viewed content areas were common injuries 74% (*n* = 54) and puberty and training 55% (*n* = 40). At the time of data collection, 45% (*n* = 33) of the initial adopters reported currently using the platform, while 40% (*n* = 29) stated they were no longer active users.

**TABLE 5:** Adoption of the digital health platform.

	Coach and parent, *n* (%)	Parent only, *n* (%)	Coach only, *n* (%)
Adoption (*N* = 73)	27 (37)	9 (12)	37 (51)
Approximately how often have you visited the health platform Frisk.Friidrott			
Daily	0 (0)	0 (0)	0 (0)
At least once a week	1 (4)	0 (0)	0 (0)
At least once a month	4 (15)	1 (11)	6 (16)
Less often but regularly	12 (44)	1 (11)	13 (35)
On some rare occasion	10 (37)	7 (78)	18 (49)
The digital health platform has several information/fact pages. Which of these do you recognize?^†^			
Training planning	12 (44)	1 (11)	15 (41)
Puberty and training	16 (59)	2 (22)	22 (59)
Recovery	11 (41)	1 (11)	14 (38)
Risk factors for injuries and how to prevent them	15 (56)	4 (44)	19 (51)
Common injuries in athletics	23 (85)	5 (56)	26 (70)
Illnesses	2 (7)	0 (0)	8 (22)
Mental health	8 (30)	1 (11)	18 (49)
Acute management	11 (41)	1 (11)	14 (38)
Safe sport	10 (37)	4 (44)	17 (46)
Antidoping	9 (33)	2 (22)	21 (57)
Are you currently using the health platform?			
Yes	16 (59)	2 (22)	15 (41)
No	9 (33)	7 (78)	13 (35)
Do not know	2 (7)	0 (0)	9 (24)

^†^Could report more than one option.

### 3.3. User Experience

Among initial adopters (*n* = 73), perceptions of usefulness (Table 6) were generally positive: 70% (*n* = 51) reported benefiting from the platform’s content, 90% (*n* = 66) rated the information as credible, and 84% (*n* = 61) indicated they encountered no obstacles when using the website. Regarding perceived ease of use (Table 7), 45% (*n* = 28) rated the layout as good or very good (scores 6–7 on the scale), 35% (*n* = 22) rated the structure as good or very good, and 44% (*n* = 27) rated user friendliness as good or very good. In total, 61% (*n* = 38) assessed the quality of the information as good or very good, and 71% (*n* = 46) stated that they would recommend the platform to others.

**TABLE 6:** Perceived usefulness of the digital health platform.

	Coach and parent, *n* (%)	Parent only, *n* (%)	Coach only, *n* (%)
Number of participants (*N* = 73)	27 (37)	9 (12)	37 (51)
Do you feel that you had any benefit/use from the content on the health platform?^†^			
Yes	24 (89)	6 (67)	21 (57)
No	1 (4)	1 (11)	0 (0)
Cannot say	2 (7)	2 (22)	15 (41)
Do you feel that the information on the health platform is credible?			
Yes	25 (93)	7 (78)	34 (92)
No	0 (0)	1 (11)	0 (0)
Cannot say	2 (7)	1 (11)	3 (8)
Have you searched for information on the health platform that you could not find on the site?			
Yes	4 (15)	1 (11)	5 (14)
No	9 (33)	2 (22)	10 (27)
Do not know	14 (52)	6 (67)	22 (60)
Do you feel that there are any obstacles for you to use the health platform?^‡^			
Yes	0 (0)	0 (0)	0 (0)
No	22 (85)	7 (88)	32 (89)
Do not know	4 (15)	1 (12)	4 (11)

^†^One coach only did not respond to the question.

^‡^One coach and parent, one parent only, and one coach only did not respond to the question.

**TABLE 7:** Perceived ease of use of the digital health.

	Likert scale	Coach and parent, *n* (%)	Parent only, *n* (%)	Coach only, *n* (%)
Number of participants (*N* = 62)		23 (37)	8 (13)	31 (50)

Rate the health platform Frisk.Friidrott from 1 (Bad), 4 (Neutral) to 7 (Very Good) regarding:				

The graphics (the layout of the page, fonts, and images)	1–3	0 (0)	1 (13)	0 (0)
	4–5	8 (35)	5 (63)	20 (65)
	6–7	15 (65)	2 (25)	11 (35)

Structure/menu (page structure with the different sections)	1–3	0 (0)	1 (13)	2 (6)
	4–5	13 (57)	4 (50)	20 (65)
	6–7	10 (43)	3 (38)	9 (29)

Information (page content)	1–3	0 (0)	0 (0)	0 (0)
	4–5	7 (30)	5 (63)	12 (39)
	6–7	16 (70)	3 (38)	19 (61)

User friendliness (how easy/difficult is it to find what you are looking for)	1–3	1 (4)	2 (25)	1 (3)
	4–5	9 (39)	4 (50)	18 (58)
	6–7	13 (57)	2 (25)	12 (39)

How likely would you recommend the health platform Frisk.Friidrott to a friend, colleague or other athlete on a scale from 1 (Extremely Unlikely), 4 (Neutral) to 7 (Extremely Likely)?^†^	1–3	0 (0)	0 (0)	0 (0)
	4–5	4 (17)	5 (63)	10 (30)
	6–7	20 (83)	3 (37)	23 (70)

^†^24 coach and parent, 8 parent only, and 33 coach only responded to the question.

Three themes based on three predefined categories and identified subcodes emerged from the analysis of the free‐text responses and semistructured interviews (Table 8). The first theme *“Use of the digital health platform”* illustrates that the platform was mainly used for knowledge acquisition and knowledge transfer, for example, as an informal educational resource and for sharing health information with parents. The second theme is *“Drivers of increased health platform engagement;”* here participants suggested that increased visibility through marketing and the inclusion of more practical, applied content could enhance adoption. Finally, the third theme “*Adopters identified needs for platform modification”* displayed that some users experienced challenges with navigation and page structure, indicating that improved organization and usability could further facilitate engagement with the platform. Quotations illustrating the themes and subcodes are presented in Table 8.

**TABLE 8:** Free‐text responses (*n* = 40) and the semistructured interviews (*n* = 5).

	Example quotes from coaches and parents
*Theme 1: Use of the digital health platform*	
Knowledge acquisition	To develop my knowledge.
	I use it sporadically, mostly as an encyclopedia.
Need access to knowledge	We have had an eating disorder that we didn’t catch. Which I feel sorry for. If we coaches had talked better, we would have understood. And if we had had more knowledge about this. It was studied afterward.
	If you could get this information earlier in coaching training, already in education for 7–10 year olds. I think it would be valuable to have this.
Informal education/teaching	Information taken from the page on short theory briefings with youth athletes.
	In connection with each training camp, we have sessions where we discuss a specific topic. It could be sleep, recovery, diet or injuries, or it could be training planning so that they understand why we train the way we do…
Training planning	It is very good because it provides planning support.
	I’ve had some athletes with some injury niggles, and when I became aware of this, I immediately thought that this should be checked on Frisk.Friidrott.
Disseminate information	I usually recommend it to others when they ask questions about rehab and injuries.
	I have also noticed a change among the parents who seem to have much higher demands today but no knowledge. And then it’s very good that they get it too. Then I refer them to Frisk.Friidrott where they get information.
	… when I had this parent‐teacher conference, I used information from Frisk.Friidrott.

*Theme 2: Drivers of increased health platform engagement.*	
Marketing via social media, newsletter	Remember it exists!
	… but I think that the federation has started with a newsletter. If you can include information there, then the clubs and districts can also see it.
Applied recommendations	Not only do I want to know more about injuries, diet or recovery in general, but I want a concrete suggestion for Schlatter rehab exercises.
	Provide a PowerPoint presentation on five slides or something or the like that you can use as a coach, with examples of things that you can actually just take from there and apply to your training group.
More concrete and practical tips	Even more material and tips based on research and experience!
	It is good to learn about pain and so on. But what is the first step? What should I do as a coach?
	I have thought about short 10‐ to 15‐min TED Talks. It is challenging for those who have to give talks to package what you want out efficiently.

*Theme 3: Adopters identified needs for platform modification*	
Web site navigation	One must learn how to navigate the site to find the right information.
	… but maybe not completely, simply. Now I don’t remember exactly how I did it when I searched, but I clicked around a bit among the headings. And then you find what you need.
Web site structure	Lots of tabs under certain headings, can you break it up in a way that makes it easier to access/find the right information faster?
	On my computer, I can’t scroll down and see the drop‐down menu, but I have to click on the facts and information heading and then scroll down the page.

*Note:* Themes with identified subcodes.

## 4. Discussion

The present study evaluated the diffusion and user experience of a digital health platform, *FriskFriidrott.se*, within the social system of Swedish Athletics. Using two dimensions from the RE‐AIM‐SSM framework—reach and adoption—this study adds to the understanding of how digital health innovations spread in a volunteer‐based, largely nonprofessional sports context. The analysis of user experience, through perceived usefulness and perceived ease of use, further highlights key facilitators and barriers to adoption. Together, the results from this study can contribute to increased understanding in the emerging field of implementation and dissemination research in sports and exercise medicine by identifying how educational, social, and perceptual factors may shape diffusion processes [12, 13, 31].

The results indicate that diffusion of the platform was modest overall, with about one‐third of respondents aware of *FriskFriidrott.se* and just over one‐quarter having initially adopted it. According to Rogers’ Diffusion of Innovation theory [16], innovations are more likely to spread when promoted by respected influencers, supported by formal communication channels, and embedded in existing community structures. In this study, knowledge of the digital platform came primarily from central communication by Swedish Athletics, rather than from peers or local clubs, demonstrating limited horizontal diffusion. This pattern underscores a need for more structured dissemination strategies, for example, incorporating information about the platform into coach education programs, newsletters, or workshops—to foster network‐level awareness and engagement [31, 32].

Initial adoption was greater among coaches than parents, particularly among those with prior education in athletics or sports medicine. This finding suggests that professional knowledge and familiarity with scientific content may facilitate adoption, a consistent conclusion across community sport research [12]. Coaches with sport‐specific education may be better positioned to recognize the platform’s credibility and relevance, while parents may need simpler, more accessible channels. Hence, tailored messages for nonspecialist user groups could improve diffusion.

Among those who adopted *FriskFriidrott.se*, perceived usefulness was rated high, reflecting strong recognition of its credibility and value. This aligns with the Technology Acceptance Model, which identifies perceived usefulness and ease of use as vital predictors of technology uptake [20, 21]. Similar relationships between user experience and sustained digital engagement have been observed in digital health research [19, 22]. Coaches mainly used the platform for education, training planning, and knowledge sharing, i.e., functions directly connected to their coaching practices and interpersonal roles.

However, perceptions of ease of use were somewhat lower, particularly regarding navigation and site structure. Such usability challenges are well‐documented in digital health programs [5] and can limit ongoing engagement even when users acknowledge the intervention’s value. Participants’ suggestions were to simplify the site architecture, clarify menus, and provide more ready‐to‐use materials, indicating that the next design iteration should prioritize participatory approaches, for example, including coaches’ identified needs and user testing to refine navigation and content presentation [33, 34].

The combination of promising effectiveness results from the earlier randomized controlled trial [7] with limited diffusion observed in this study reflects a common research–practice gap in sports medicine. This gap, frequently highlighted in implementation literature, arises when effective interventions are left to diffuse naturally, without structured support [9, 35, 36]. Within the decentralized, volunteer‐driven structure of Swedish Athletics [25, 37], contextual barriers, such as lack of licensure, varied club resources, and diffuse communication networks, further restrict diffusion.

Effective strategies from the national sports federation will therefore require embedding areas from the platform into formal systems, such as coach education curricula. Learning modules developed on the platform can be tailored to different levels of coach training and include both self‐study materials (e.g., videos and readings) and lectures [31, 32]. Continuous promotion of the platform through federated channels and social media is a way to reach a wider audience [31, 32]. Co‐creation with end users has also been shown to enhance adoption and long‐term integration of innovations [33, 34]. By embracing these principles, *FriskFriidrott.se* could achieve greater reach and sustainability within Swedish Athletics. Future research may also examine how emerging tools such as generative AI influence how coaches and parents seek and evaluate health information beyond federation‐based platforms.

This study possesses several notable strengths. It is among the first to examine the diffusion of a digital health innovation within a volunteer‐based sport federation, applying established implementation frameworks such as RE‐AIM‐SSM and the Technology Acceptance Model. The multimethods design allowed for triangulation between quantitative indicators of diffusion and qualitative insights into user experience, thereby strengthening the validity and depth of interpretation. Data collection and reporting followed recognized quality standards (STROBE and COREQ), ensuring methodological transparency and reproducibility. The sample included both parents and coaches, two key stakeholder groups in youth athletics, which provided an ecological understanding of diffusion processes across the Swedish Athletics system. Finally, the integration of theoretical perspectives from implementation science and digital health offers practical relevance for other sports and community‐based health initiatives.

However, the study also has limitations. The cross‐sectional design captures diffusion and user experience at a single point in time, limiting causal inference and the ability to assess long‐term trends. Conducting research in smaller individual sports presents various challenges, one is to reach and recruit participants [38]. The primary limitation of this study is the relatively small sample size, which may have reduced statistical power and limited the precision of our estimates. Moreover, the total population of athletes and coaches in Swedish athletics is unknown, as no central registers exist at any competitive level. This precludes the calculation of response rates and hampers the assessment of the sample’s representativeness relative to the underlying target population. Compounding this issue, the various recruitment strategies employed—namely, outreach through the Elite Clubs Association, dissemination via social media, and distribution through the coach education network—relied on voluntary participation, which may have introduced selection bias and limited the representativeness of the sample. Specifically, these strategies may have disproportionately included individuals already engaged in health‐related initiatives or with prior experience in research collaborations. They may also have overrepresented individuals with greater digital literacy, higher motivation to engage with injury prevention or health promotion initiatives, or stronger existing connections to athletics organizations. This potential overrepresentation of health‐aware respondents should be carefully considered when interpreting the generalizability of the findings.

Furthermore, participation in organized sports in Sweden tends to be skewed toward families of higher socioeconomic status. Consequently, both the recruitment process and the observed outcomes may be influenced by underlying differences in health behaviors and living conditions across socioeconomic strata. As a result, the findings of this study should be considered context‐dependent and interpreted with caution when generalized to other age groups, sporting contexts, or cultural settings beyond Sweden and the broader Scandinavian region. Regarding measurement, the survey instrument, while adapted from validated measures and revised for the athletics context, was not specifically validated within that context. With only a small number of omitted responses among the 267 participants, we consider the survey to have been well understood by the respondents. For the qualitative component, the relatively small number of interviews and free‐text responses constrains transferability of the findings beyond this sample. Additionally, because the platform was evaluated after largely passive dissemination, the observed reach and adoption rates may underestimate its potential under more active implementation conditions. Conversely, the self‐selected nature of the sample may also have led to an overestimation of awareness, interest, or adoption among the broader athletics population. Despite these limitations, the present study contributes valuable insights into the diffusion processes of a digital health platform in a sports context and calls for larger, more representative studies to verify the current observations.

## 5. Conclusions

Diffusion of the *FriskFriidrott.se* digital health platform within Swedish Athletics was modest and primarily limited to coaches with prior education and sports medicine experience. Adoption among parents remained low, indicating that passive diffusion alone is insufficient to ensure equitable reach across stakeholder groups. Adopters recognized the platform’s credibility and usefulness, but ease of use and structural aspects of the website require improvement.

Bridging the research–practice gap in youth athletics requires not only evidence‐based content but also systematic engagement with the social and organizational contexts in which the innovation is intended to function. To enhance sustainable use and maximize the health‐promoting potential of the platform, future efforts should focus on active and structured dissemination and implementation strategies, including incorporation into formal coach education programs and use of social media and newsletters for ongoing promotion.

## Author Contributions

Jenny Jacobsson and Siw Carlfjord conceived and designed the research project. Jenny Jacobsson coordinated the development of the study. Jenny Jacobsson and Örjan Dahlström were involved in data collection. Jan Kowalski performed the statistical analysis. Jenny Jacobsson and Ulrika Tranaeus were involved in the analysis of the qualitative data. All authors made substantial contributions to data interpretation. Jenny Jacobsson and Per Nilsen made a substantial contribution to drafting and writing the article. All authors were involved in revising the manuscript. Jenny Jacobsson is the guarantor of the study.

## Funding

The study received research support from the Folksam Research Foundation, Stockholm, Sweden.

## Disclosure

Participants were not involved in the design, reporting, or dissemination of the research. All authors approved the final the version of the manuscript to be published. The supporting federation has not been involved in data analysis or interpretation of data.

## Ethics Statement

This study involves human participants. The Swedish Ethical Review Authority (Dnr 2023‐02068‐1) assessed the study and declared that studies like these are exempt from the national laws of health research and do not need to apply for ethical approval (Sections 3 and 4 of the Swedish Ethics Review Act). The study was conducted in compliance with the Declaration of Helsinki. All participants were informed about the study beforehand and gave their informed consent to participate.

## Consent

Consent was obtained directly from the participants.

## Conflicts of Interest

Jenny Jacobsson is the medical coordinator at the Department for Elite and National Team at Swedish Athletics. The other authors declare no conflicts of interest.

## Supporting Information

Additional supporting information can be found online in the Supporting Information section.

## Supporting information


**Supporting Information** Filename: Diffusion_Supporting information_TSM.pdf. Description: Supporting Information Additional supporting information (Supporting Information) to this article can be found in the Supporting Information section.

## Data Availability

The data that support the findings of this study are available from the corresponding author upon reasonable request.
