# Supplementary material for: Bridging the Research–Practice Gap: Diffusion and User Experience of a Digital Health Platform in Swedish Athletics
Source: Transl Sports Med. 2026 Sep 30;2026:2452118. doi: 10.1155/tsm2/2452118 (PMC13624697; doi:10.1155/tsm2/2452118)
Supplement: Supplementary file 1 [file TSM2-2026-2452118-s001.zip › Diffusion_Supplemental material_TSM.pdf]

Supporting Information

Bridging the Research–Practice Gap: Diffusion and User Experience of a Digital Health Platform in Swedish Athletics

Jenny Jacobsson,[1,2,3] Per Nilsen,[1] Siw Carlfjord,[1] Jan Kowalski,[3] Örjan Dahlström,[2,4] Ulrika Tranaeus[5,6]

Appendix A. Questionnaire used for data collection 2

Appendix B. Semi-structured interview guide…………………………………………………………….5

Appendix A. Questionnaire used for data collection

**Questions**	**Response options**
**Baseline questionnaire**	
I participate as	a. coach
	b. parent/caregiver
	c Both coach and parent
I identify myself as a:	Man/woman/ do not wish to state
Age	Pulldown menu with options from 15 to 65 years
Were you born in	a. Sweden
	b. Norway / Finland / Denmark or Iceland
	c. Another country in Europe
	d. Other country outside Europe
Have you been an athlete yourself?	a. Yes. If yes, in which sport/sports have you been most active in?
	b. No
Are you a coach today?	Yes/no, If yes, enter the sport (free text)
In which sport do you do your main coaching job?	a. Athletics
	b. Other sport (free text)
Have you taken any coach courses in athletics?	Yes/no. If yes which ones (various options provided and free text)
Does your club/district offer any form of sports medicine and/or health training for coaches and parents in athletics?	Yes/no/do not know. If yes what kind of education?
Have you attended any training in sports medicine?	Ex tape course, emergency care? Other? Yes/no/do not know
If you are looking for information about, for example, injuries in athletics, emergency care or diet, how do you do it? (You can specify more options)	a. Look for information online
	b. Ask other coaches or parents
	c. Contact your health centre
	d. Contact with another doctor/physiotherapist
	e. Otherwise? Please describe
**Questions about the digital health platform Frisk.Friidrott**	

Have you previously heard of the digital health platform Frisk.Friidott?	Yes/no. If yes, where did you learn about the digital platform?<br>a. Swedish Athletics Federation<br>b. My district<br>c. My club<br>d. Other coaches<br>e. Other parents<br>f. Doctor, physiotherapist<br>g. Other, please specify (free text option)
Have you visited the health platform Frisk.Friidrott? Yes/no. If yes, approximately how often?	a. Daily<br>b. At least once a week<br>c. At least once a month<br>d. Less often but regularly<br>e. On a rare occasions
The digital health platform has several information/fact pages. Which of these do you recognize?	a. Training planning<br>b. Puberty and exercise<br>c. Recovery<br>d. Causes of and how injuries are prevented<br>e. Common injuries in athletics<br>f. Diseases<br>g. Mental health<br>h. Emergency care<br>i. Safe sport<br>j. Anti-doping
**Questions for those who have visited the health platform to know a little about your personal impressions of it**	
Do you feel that you had any benefit/use from the content on the health platform?	Yes/no/cannot say. If yes, please describe how you have used the digital platform. (free text option)
Do you feel that the information on the health platform is credible?	Yes/no/cannot say
Have you searched for information on the health platform that you could not find on the site?	Yes/no/do not know

Do you feel that there are any obstacles for you to use the health platform?	Yes/no/do not know
Are you using the health platform today?	Yes/no/do not know. If yes, please describe how you use the digital platform today. (free text option). If no, please describe what would make you use the health platform more. (free text option)
**If you have visited the digital health platform, you can help us rate it**	
Rate the health platform Frisk.Friidrott from poorest to highest regarding the graphics (the layout of the page, fonts and images),	1 (bad), 4 (neutral), 7 (very good)
Rate the health platform Frisk.Friidrott from poorest to highest regarding structure/menu (page structure and the different sections)	1 (bad), 4 (neutral), 7 (very good)
Rate the health platform Frisk.Friidrott from poorest to highest regarding information (page content)	1 (bad), 4 (neutral), 7 (very good)
Rate the health platform Frisk.Friidrott from poorest to highest regarding user friendliness (how easy/difficult is it to find what you are looking for)	1 (bad), 4 (neutral), 7 (very good)
Is there anything you would like to change regarding the site (the content of the page)?	Free text
Would you recommend the health platform Frisk.Friidrott to a friend, colleague or other athlete?	0 (extremely unlikely) to 7 (extremely likely)
Anything else you would like to comment on, provide feedback on? Feel free to write!	Free text
To increase our understanding of how the digital health platform is experienced by those of you who used/use it, we would like to conduct some interviews in the winter of 2023/2024 with some of you who participated in the study. If you are interested in participating in an interview study, leave an email address here	

The Swedish to English translation of the questions/response options in Appendix A was not done using a validated method.

Appendix B: Interview guide

Interview guide: Frisk reach and adoption

Before the interview begins, participants' consent is collected. The participant is also reminded of confidentiality, that the data material is pseudonymized in the study, and they have the right to skip questions or end the interview at any time.

Background questions:

- How old are you?
- In which function/role are you participating in the study? (coach, parent, other)

Initial questions:

- Can you briefly tell us about your own sporting background?
- Can you briefly tell us about your current assignment in athletics?
- Do you have any education in sports medicine/health?
- Based on your current role in athletics, how have you experienced health among young athletics? (positive effects, negative effects such as injuries)

The participant's general reflections on seeking information about health:

- If you are looking for information about health problems (in athletics), for example injuries, how do you do it (e.g. Google, talk to other coaches, parents)?
- Tell us about websites on the Internet that you know or usually visit to get answers to questions about health related to athletics (why that page(s))?
- What health information do you think coaches and parents of young athletics need to have access to (why is this information important)?

The participant's reflections on using the Frisk health platform:

- What experience do you have of using the platform (for what purposes it has been used)?

- Is there anything on the platform that you found particularly valuable (please describe in more detail and why)?
- Are there any particular areas/sections you visited more than once (why these particular sections)?

End of interview:

- What do you think of the health platform layout? Is it easy to find what you are looking for (experience, language, logical headings, etc.)?
- What would make you use the health platform (start using it or use it more)?

After completing the interview, the participant is thanked for their participation. The participant also receives information about where can take part in future results of the study.

The Swedish to English translation of the interview guide in Appendix B was not done using a validated method.
