# Supplementary material for: Bridging the Research–Practice Gap: Diffusion and User Experience of a Digital Health Platform in Swedish Athletics
Source: Transl Sports Med. 2026 Sep 30;2026:2452118. doi: 10.1155/tsm2/2452118 (PMC13624697; doi:10.1155/tsm2/2452118)

Supplement Content: A COREQ flow-chart showing the recruitment of participants

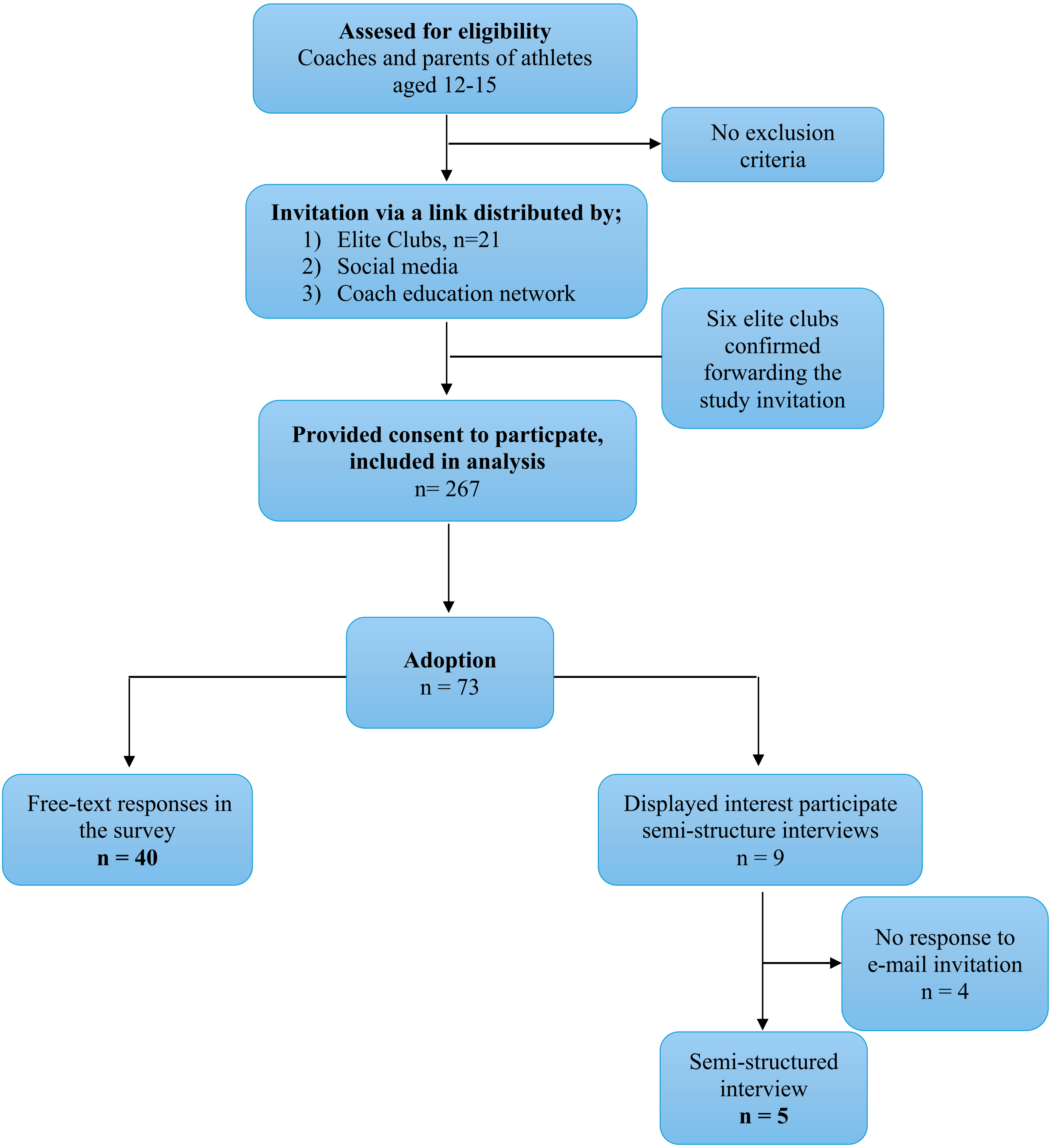

Supplement: Supplementary file 1 [file TSM2-2026-2452118-s001.zip › Supplement Content_Flow chart_COREQ.pdf]
